# Elderly Woman With Abdominal Pain and Distension

**DOI:** 10.1016/j.acepjo.2026.100398

**Published:** 2026-04-18

**Authors:** Brian Quach, Andrew Eyre

**Affiliations:** 1 Frank H. Netter MD School of Medicine at Quinnipiac University, North Haven, Connecticut, USA; 2 Department of Emergency Medicine, Brigham and Women’s Hospital, Harvard Medical School, Boston, Massachusetts, USA

**Keywords:** Ogilvie syndrome, abdominal pain, acute abdomen, colonic ileus, acute colonic pseudo-obstruction

## 1 Patient Presentation

**Figure 1:**
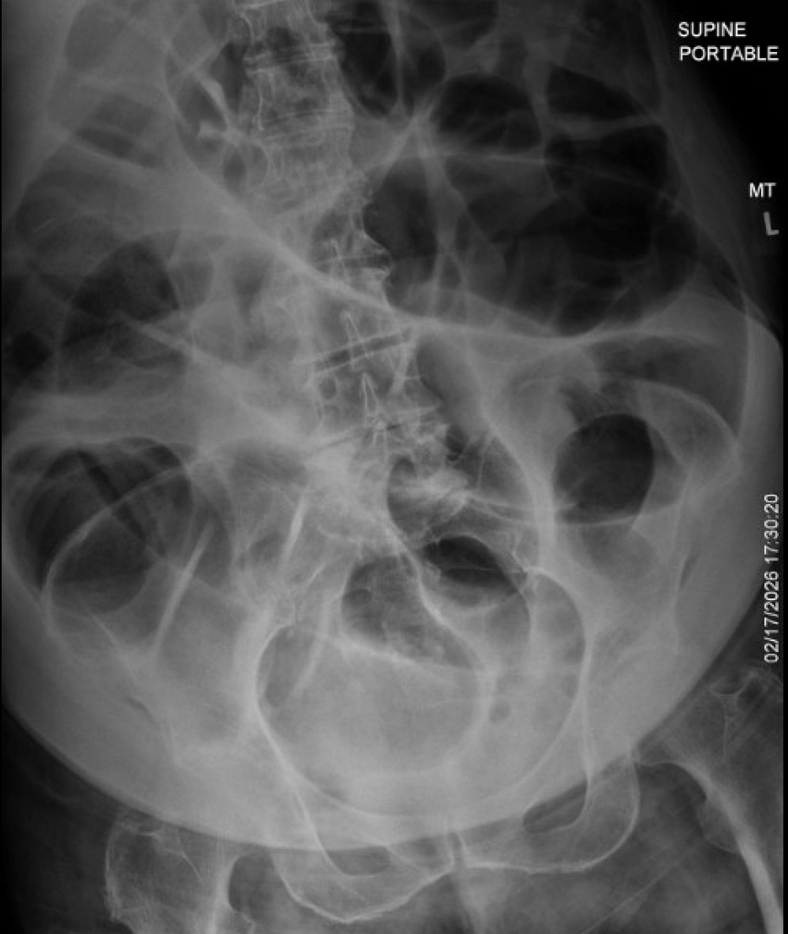
Supine abdominal radiograph obtained at time of emergency department presentation. Dilated gas-filled loops of large bowel, measuring up to 10.8 centimeters.

**Figure 2:**
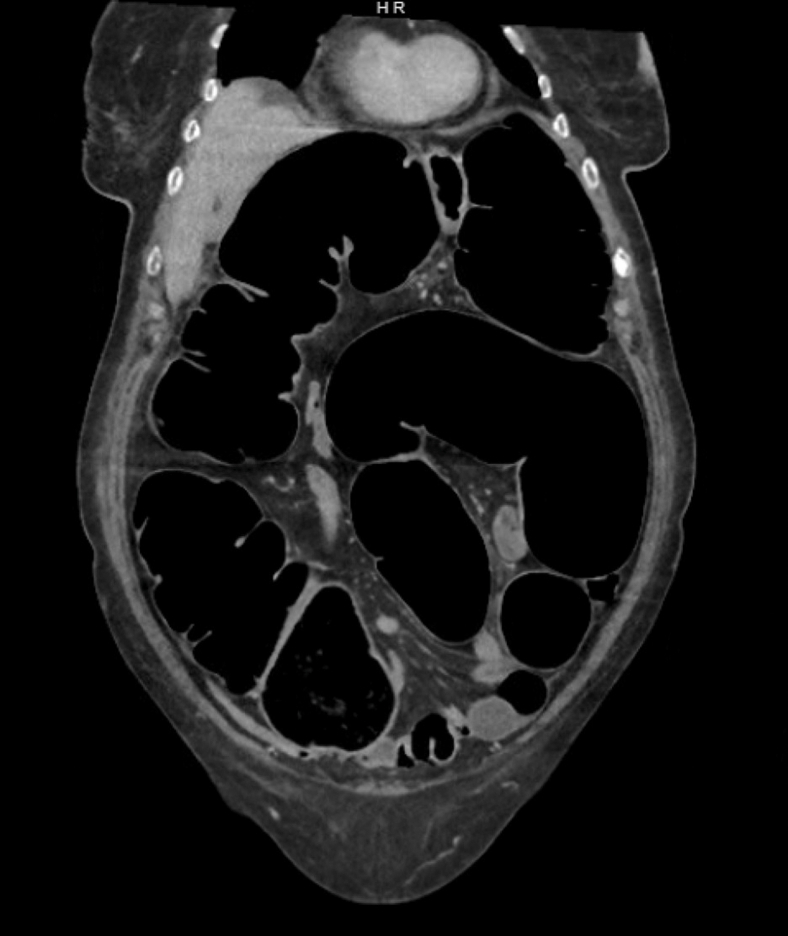
Coronal computed tomography scan revealing diffuse distention of abdominal cavity demonstrating marked dilation of large bowel structures.

**Figure 3:**
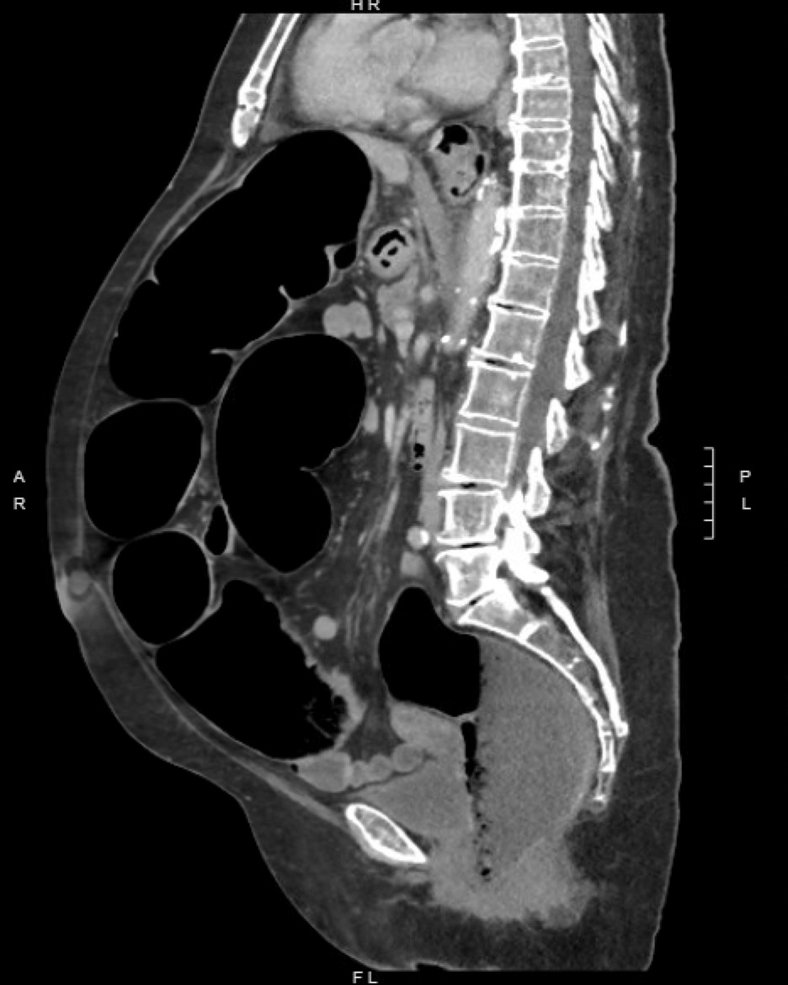
Sagittal view of computed tomography abdomen/pelvis revealing distention of large bowel.

**Figure 4:**
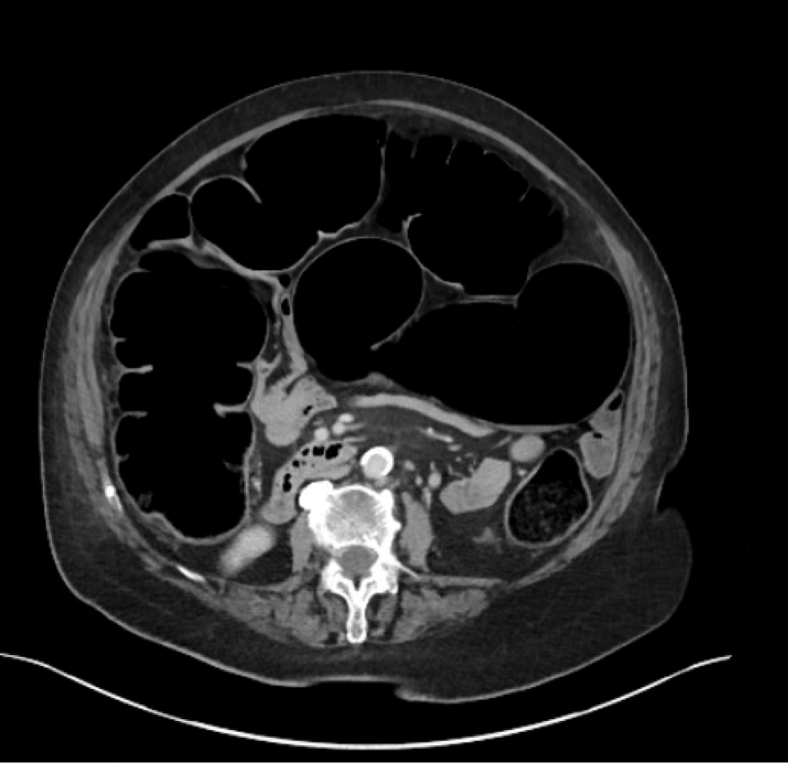
Axial computed tomography scan revealing marked distention of large bowel gas-filled with air-fluid levels and normal appearing appendix.

An 80-year-old woman with a history of hypothyroidism and cholecystectomy performed 4 months prior presented to the emergency department with a 2-day history of progressive abdominal pain and distention. Vital signs were significant for a blood pressure of 147/87 mmHg. Abdominal examination was remarkable for significant distention, tympany to percussion, and diffuse tenderness to palpation. The skin was warm and dry, negative for jaundice. Laboratory testing revealed hypokalemia. A bedside radiograph (Fig 1) and computed tomography scan (Figure 2, Figure 3, Figure 4) were obtained. The surgery team was consulted, and intravenous (IV) fentanyl was administered for analgesia prior to admission.

## 2 Diagnosis: Ogilvie Syndrome

Ogilvie syndrome is a rare medical condition that involves severe large bowel distention without a notable obstruction or infection.^1^^,^^2^ The clinical presentation may resemble acute colorectal obstruction with megacolon from gas buildup due to parasympathetic hyperactivity that occurs following trauma-induced abdominal injury or electrolyte disturbances, particularly, hypocalcemia and hypokalemia.^1^^,^^2^ If left untreated, this condition may lead to bowel perforation.^1^^,^^3^

In the emergency department, Ogilvie syndrome may be managed in a conservative manner when the clinical suspicion for perforation is low.^1^^,^^4^ Literature supports the use of neostigmine as an initial treatment option prior to considering invasive maneuvers such as colonoscopic decompression or surgery.^4^, ^5^, ^6^ The patient responded well to IV neostigmine resulting in symptomatic relief and fecal disimpaction. Additional supportive measures included IV hydration with lactated ringers, potassium, and ondansetron for antiemetic therapy.

## Conflict of Interest

Author Eyre serves as an adviser to MedVR Education and Apoqlar, extended-reality medical education companies. Their products are not discussed in this paper.

## References

[1] Pereira P., Djeudji F., Leduc P., Fanget F., Barth X. (2015). Ogilvie's syndrome–acute colonic pseudo-obstruction. J Visc Surg.

[2] Haj M., Haj M., Rockey D.C. (2018). Ogilvie's syndrome: management and outcomes. Medicine (Baltimore).

[3] Tenofsky P.L., Beamer R.L., Smith R.S. (2000). Ogilvie syndrome as a postoperative complication. Arch Surg.

[4] McNamara R., Mihalakis M.J. (2008). Acute colonic pseudo-obstruction: rapid correction with neostigmine in the emergency department. J Emerg Med.

[5] Ponec R.J., Saunders M.D., Kimmey M.B. (1999). Neostigmine for the treatment of acute colonic pseudo-obstruction. N Engl J Med.

[6] Peker K.D., Cikot M., Bozkurt M.A. (2017). Colonoscopic decompression should be used before neostigmine in the treatment of Ogilvie’s syndrome. Eur J Trauma Emerg Surg.

